# Orphan nuclear receptor Nur77 inhibits poly (I:C)-triggered acute liver inflammation by inducing the ubiquitin-editing enzyme A20

**DOI:** 10.18632/oncotarget.17731

**Published:** 2017-05-09

**Authors:** Xiu-Ming Li, Tian-Yu Yang, Xiao-Shun He, Jing-Ru Wang, Wen-Juan Gan, Shen Zhang, Jian-Ming Li, Hua Wu

**Affiliations:** ^1^ Pathology Center and Department of Pathology, Soochow University, Suzhou 215123, China; ^2^ Department of Pathology, The First Affiliated Hospital of Soochow University, Soochow University, Suzhou 215006, China

**Keywords:** Nur77, liver inflammation, NF-κB, A20, mouse model

## Abstract

Inflammation is a key contributor to various types of acute and chronic liver disease. We recently reported that lack of Nur77, an orphan nuclear receptor, contributes to the pathogenesis of inflammatory diseases including inflammatory bowel disease and sepsis. However, whether Nur77 plays a critical role in liver inflammation remains to be fully understood. Employing *in vivo* acute liver inflammation model in wild-type (Nur77^+/+^) and Nur77^-/-^ mice, we here found that Nur77 deficiency dramatically increased the production of pro-inflammatory cytokines and accelerated liver injury induced by poly (I:C)/D-GalN in Nur77^-/-^ mice. Mechanistically, Nur77 acts as a negative regulator of NF-κB signaling by inducing the expression of ubiquitin-editing enzyme A20, a novel target gene of Nur77. Notably, in inflammatory cells, overexpression of A20 enhanced, whereas knockdown of A20 by siRNA approach impaired, the inhibitory effect of Nur77 on poly (I:C)-triggered inflammation. Collectively, our data suggest that the orphan nuclear receptor Nur77 plays a protective role in poly (I:C)-triggered liver inflammation by inducing A20, thus making it a promising target for the prevention and treatment of liver inflammation.

## INTRODUCTION

Inflammation contributes to the pathogenesis of acute and chronic liver diseases induced by different etiologies, such as drug toxicity and viral infection [1]. Excessive inflammatory response in the liver can lead to liver injury, which destroys liver function in the maintenance of homeostasis [2, 3]. In absence of liver inflammation, the intrahepatic inflammatory cells including macrophages, monocytes, and natural killer (NK) cells remains roughly constant and activity levels are normal [1]. However, strong liver inflammation results in sudden increased recruitment of inflammatory cells to the liver to initiate and drive inflammatory response by producing proinflammatory cytokines and chemokines, such as tumor necrosis factor alpha (TNFα), interleukin-6 (IL-6), interferon beta (IFN-β) and monocyte-chemoattractant protein-1 (MCP-1) [1]. These inflammatory mediators can led to hepatocytic cell death through activation of cell death signaling [1]. Despite the great effort that has been made to understand the role of inflammation in acute and chronic liver diseases, the molecular mechanism underlying liver inflammation is not completely understood.

Nur77 (also called NR4A1, TR3, or NGFI-B) belongs to the NR4A subfamily of nuclear receptors and plays important roles in the regulation of a wide array of biological processes, including cell growth, differentiation, and apoptosis [4–6]. Recent studies have implicated Nur77 in inflammatory and immune diseases. In particular, Nur77 has been demonstrated to play a protective role in atherosclerosis, sepsis, and airway inflammation [7–9]. In experimental autoimmune encephalitis (EAE), Nur77 expression in infiltrating monocytes and derived macrophages plays a protective role in EAE, while Nur77 loss leads to accelerated and exacerbated EAE in mice [10]. Recently, data from GWAS-based analysis and *in vitro* and *in vivo* studies suggested that loss of Nur77 contributes to the pathogenesis of inflammatory bowel disease (IBD) [11]. Genetic variants of Nur77 in patients with ulcerative colitis (UC) and Crohn’s disease (CD) are associated with low Nur77 expression, which can render patients susceptible to colitis [11]. Nur77 is also shown to be important to regulatory T cell (Treg) development. Mice that are lacking all Nr4a receptors, including Nur77, suffer lethal systemic autoimmunity [12].

Recent studies have provided important insight into molecular mechanisms underlying Nur77 action in inflammatory and immune diseases. Nur77, like other members of nuclear receptor, functions in the nucleus as a transcriptional factor to regulate its target genes expression. For example, Nur77 inhibits norepinephrine (NE) production by recruiting the corepressor CoREST to the promoter of tyrosine hydroxylase (TH) gene, which is critical to Nur77’s protective role in autoimmune encephalomyelitis [10]. Nur77 suppresses endothelial inflammation by binding directly to IκBα promoter to induce expression [13]. Nur77 also has extranuclear effects that regulate some inflammatory diseases. For example, cytoplasmic Nur77 interacts with TRAF6 and can prevent TRAF6's auto-ubiquitination and oligomerization and so suppress NF-κB activation and pro-inflammatory cytokine production [11]. Disruption of this interaction in mice lacking Nur77 led to acceleration of inflammatory bowel disease [11]. In this way, these studies suggest that Nur77 can exert its nuclear or cytoplasmic action to regulate the development and progression of inflammatory diseases. The role and molecular mechanism of Nur77 in liver inflammation awaits further investigation.

A20, also known as TNFAIP3 (tumor necrosis factor alpha-induced protein 3), has been shown to have the activity of deubiquitinating enzyme (DUB) and functions as a negative regulator of inflammatory signaling NF-κB mainly through removing ubiquitin chains from NF-kB essential transducer TRAF6 [14, 15]. Several molecules have been identified to regulate A20 expression by epigenetic, transcriptional and post-transcriptional mechanisms. For examples, histone methyltransferase Ash1l enhances A20 expression through inducing H3K4 methylation at the A20 promoter [16]. Orphan Nuclear Receptor ERRα binds to A20's promoter region and transcriptionally upregulates its expression [17]. The RNA-binding protein RC3H1 inhibits A20 expression through binding to A20 3'UTR [18]. However, it is unknown whether and how orphan nuclear receptor Nur77 is involved in the regulation of A20 expression.

In this study, we assessed the essential protective role of the orphan nuclear receptor Nur77 in poly (I:C)-induced acute liver inflammation. Nur77 induced A20 expression by binding to its promoter, and it subsequently inhibited NF-κB activity and so limited poly (I:C)-induced acute liver inflammation. This study not only reveals the important role of Nur77 in liver inflammation, but also provides potential targets for future therapeutic interventions.

## RESULTS

### Nur77-knockout (Nur77^-/-^) mice show increased susceptibility to poly (I:C)/D-GalN-induced acute liver inflammation

**Figure 1:**
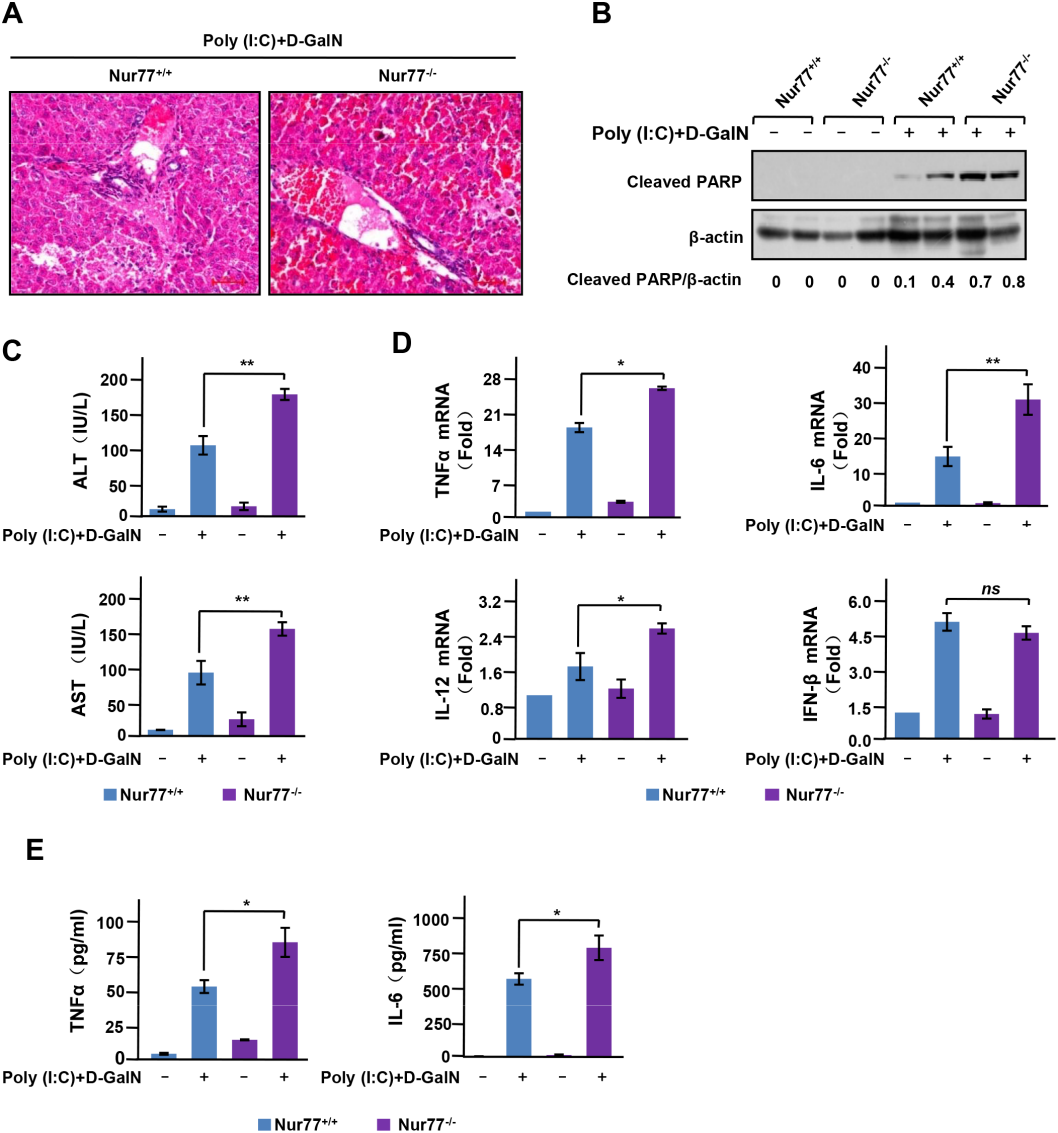
Nur77 attenuates poly (I:C)-induced acute liver inflammation **(A)** H&E staining of liver from Nur77^+/+^ and Nur77^−/−^ mice 5 h after injection of poly (I:C) (6.25 mg/kg) and D-GalN (0.5 g/kg). Representative images are shown. Scale bars, 100 μM. Original magnification, × 100. **(B)** Liver extracts were examined by Western blot analysis with antibody to cleaved PARP. Western blot analyses were quantified via densitometry, and the mean ratios of the indicated protein from three independent experiments are shown at the bottom of the figure. **(C)** ELISA assay of serum transaminase activity at 5 h after injection of poly (I:C)/D-GalN in Nur77^+/+^ and Nur77^−/−^ mice. Error bars represent mean ± s.d. *p < 0.05. **(D)** Expression of TNFα, IL-6, IL-12 and IFN-β mRNA was assessed by qPCR in the livers of Nur77^+/+^ and Nur77^−/−^ mice 3 h after treatment with poly (I:C)/D-GalN. Error bars represent mean ± s.d. from n=3 biological replicates. *P < 0.05. **(E)** ELISA of the production of TNFα and IL-6 in serum from Nur77^+/+^ and Nur77^−/−^ mice after intraperitoneal injection of poly (I:C)/D-GalN for 5 hours. *P < 0.05.

To explore the biological function of Nur77 in liver inflammation, we induced acute liver inflammation in mice by injection of poly (I:C)/D-GalN. These Nur77^-/-^ mice exhibited a significant increase in inflammatory infiltrates in hepatocytes and severe hepatocyte destruction not observed in wild-type control mice (Figure 1A). poly (I:C)/D-GalN injection also induced significant hepatocyte cell death in Nur77^-/-^ mice, as indicated by PARP cleavage (Figure 1B). Consistently, Nur77^-/-^ mice showed a more exaggerated elevation of serum alanine transaminase (ALT) and aspartate transaminase (AST) than wild-type mice after poly (I:C)/D-GalN injection (Figure 1C), indicating severe liver injury in Nur77^-/-^ mice. Because proinflammatory cytokines are critical pathological mediators of various inflammatory and immune diseases, including acute liver inflammation [19], we used qPCR to assess the expression of some proinflammatory cytokines in the liver. As shown in Figure 1D, there was more expression of TNFα, IL-6, and IL-12 mRNA in liver tissues prepared from Nur77^-/-^ mice than in those from wild-type mice, while the expression of interferon-β (IFN-β) was unchanged. Enhanced production of proinflammatory cytokines by poly (I:C)/D-GalN in Nur77^-/-^ mice was also confirmed by our measurement of levels of TNFa and IL-6 in serum from animals (Figure 1E). Taken together, these data indicate that Nur77 prevents the development of poly (I:C)-induced acute liver inflammation.

### Nur77 attenuates NF-κB activation in macrophages and monocytes

**Figure 2:**
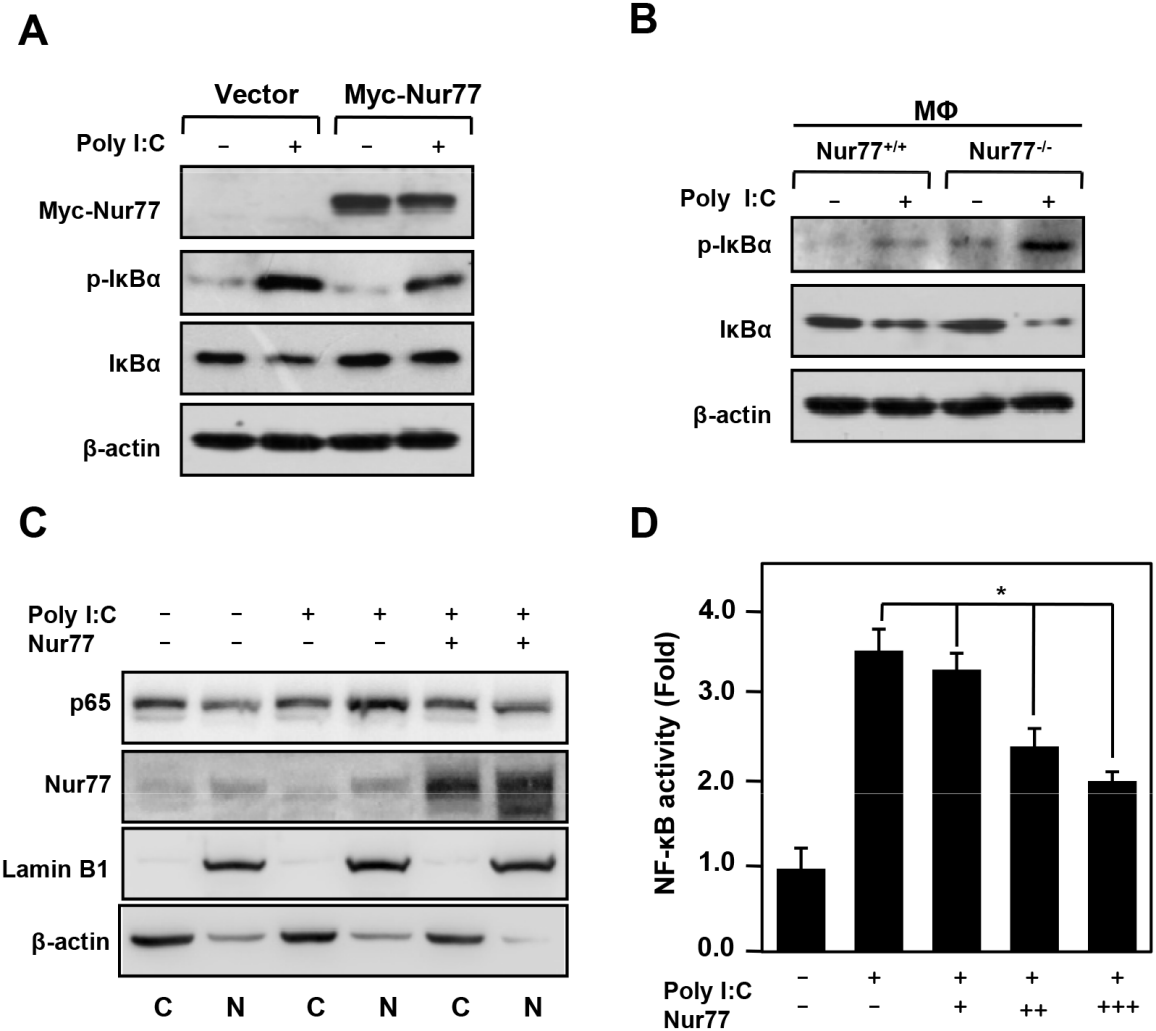
Nur77 inhibits poly (I:C)-induced NF-κB activation **(A, B)** Western blot analysis of the indicated protein in Nur77-overexpressed RAW264.7 cells **(A)** or Nur77^+/+^ and Nur77^−/−^ peritoneal macrophages (MΦ) **(B)** treated with vehicle or poly (I:C) (20 μg/ml). **(C)** Subcellular fractionation analysis of p65 expression in vehicle or poly (I:C)-treated RAW264.7 cells. Western blotting of β-actin and Lamin B1 served as controls for the purity of cytoplasmic (C) and nuclear (N) fractions, respectively. **(D)** NF-κB luciferase reporter activity was measured in vehicle and poly (I:C)-treated RAW264.7 cells.

Our recent work demonstrated that inflammatory signaling of NF-κB is responsible for the pathogenesis of inflammatory bowel disease (IBD) and hepatocellular carcinoma (HCC) [11, 20]. We next addressed whether Nur77 attenuates poly (I:C)-induced acute liver inflammation by regulating NF-κB activation. Interestingly, these results showed that overexpression of Nur77 greatly impaired poly (I:C)-induced phosphorylation and degradation of IκBα (Figure 2A). However, peritoneal macrophage from Nur77^-/-^ mice showed more phosphorylation and degradation of IκBα after poly (I:C) treatment than the peritoneal macrophage from Nur77^+/+^ mice (Figure 2B). Consistently, Nur77 significantly inhibited nuclear translocation of p65 induced by poly (I:C) (Figure 2C). Overexpression of Nur77 markedly suppressed NF-κB activation induced by poly (I:C) in a dose-dependent way (Figure 2D), further supporting the conclusion that Nur77 negatively regulates the NF-κB inflammatory signaling pathway.

### Effect of Nur77 on the expression of NF-κB downstream target genes

**Figure 3:**
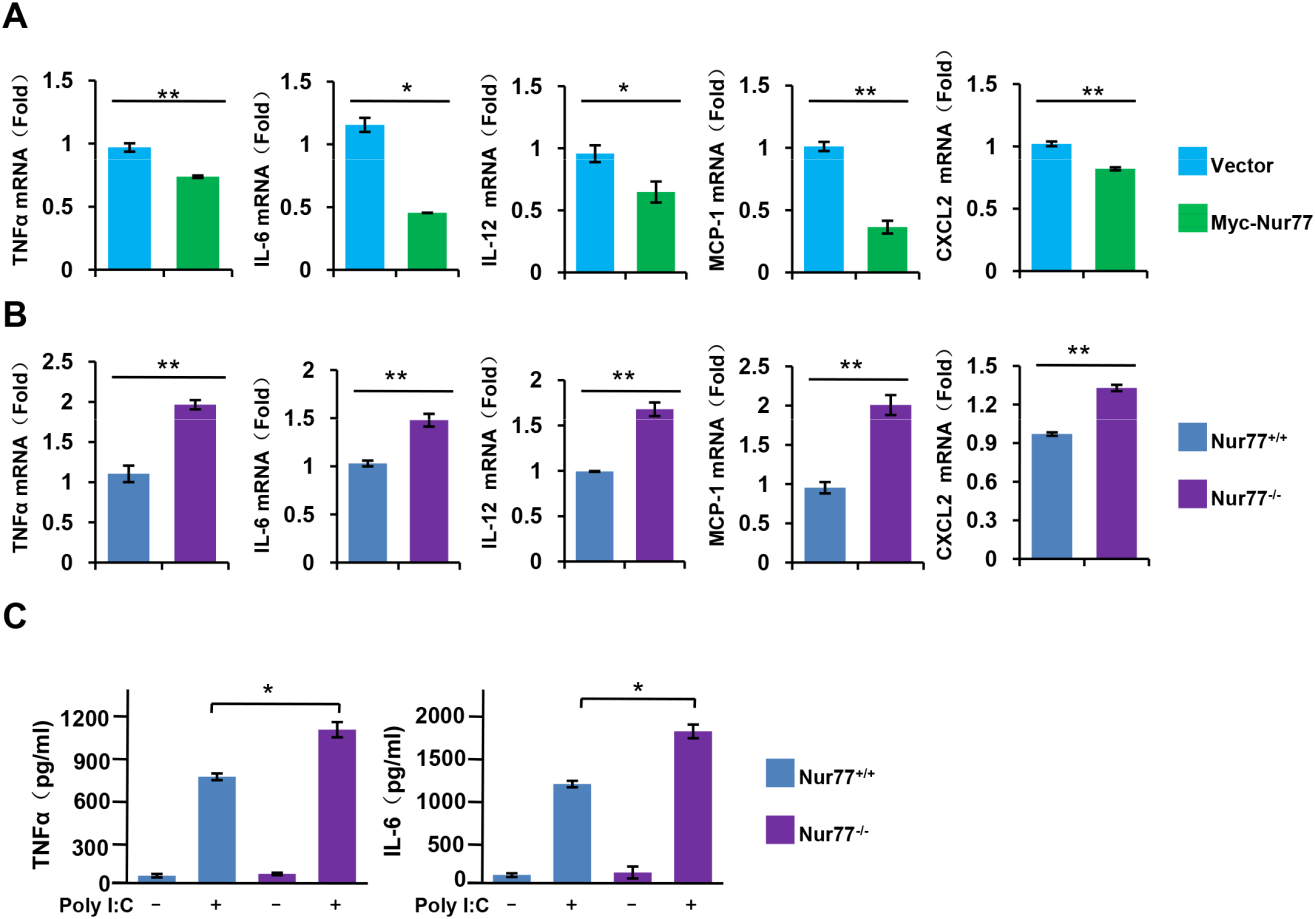
Nur77 inhibits proinflammatory cytokine expression and production *in vitro* **(A, B)** qPCR assay of the indicated cytokine mRNA expression in **(A)** WT and Nur77-transfected RAW264.7 cells, or in **(B)** Nur77^+/+^ and Nur77^−/−^ peritoneal macrophages (MΦ) after 3 h of treatment with poly (I:C) (20 μg/ml). **(C)** ELISA assay of TNFα and IL-6 in supernatants of Nur77^+/+^ and Nur77^−/−^ peritoneal macrophages (MΦ) incubated for 24 hours with poly (I:C) (20 μg/ml). *P < 0.05 and **P < 0.01.

Upon activation of NF-κB signaling, phosphorylated IκBα protein are polyubiquitinated and degraded by proteasome, allowing NF-κB to rapidly move into the nucleus and so regulate the expression of its target genes, including multiple inflammatory cytokines [21]. Using qPCR and ELISA assay, we observed that overexpression of Nur77 in RAW264.7 cells largely impaired the effect of poly (I:C) on inducing the expression of inflammatory cytokines and chemokines, including TNFα, IL-6, IL-12, MCP-1, and CXCL2 (Figure 3A), whereas peritoneal macrophages from Nur77^-/-^ mice exhibited enhanced expression and production of the cytokines and chemokines (Figure 3B, 3C). These data demonstrate that Nur77 attenuates the expression of NF-κB downstream target genes by inhibiting NF-κB activity.

### Nur77 transcriptionally enhances A20 expression

**Figure 4:**
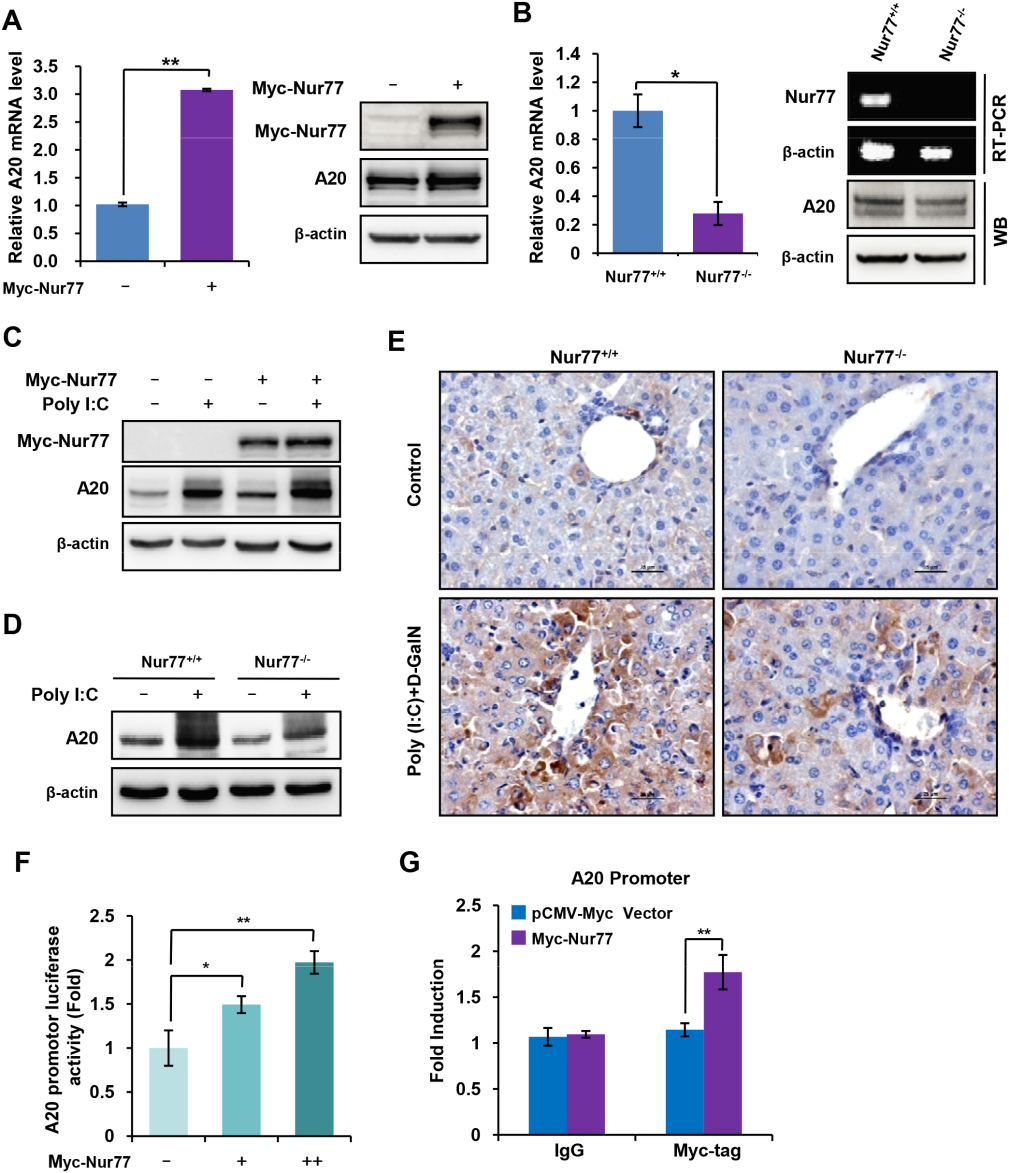
Nur77 transcriptionally enhances A20 expression in monocytes and macrophages **(A)** qPCR assay (left) or Western blot analysis (right) of A20 expression in Nur77-overexpressing RAW264.7 cells. **(B)** qPCR assay (left) or Western blot analysis (right) of A20 expression in peritoneal macrophages from Nur77^+/+^ or Nur77^-/-^ mice. **(C, D)** Western blot analysis of the indicated protein in Nur77-overexpressed RAW264.7 cells **(C)** or Nur77^+/+^ and Nur77^−/−^ peritoneal macrophages (MΦ) **(D)** treated with vehicle or poly (I:C) (20 μg/ml). **(E)** Immunohistochemical staining of A20 in the liver tissues from of wild-type mice and Nur77^-/-^ mice after poly (I:C)/D-GalN injection. **(F)** The luciferase activity of A20 promoter was measured in RAW264.7 cells transfected with the increasing amount of Nur77. **(G)** Binding of Nur77 to the A20 promoter by chromatin immunoprecipitation (ChIP) assay. ChIP assays were performed using anti-myc tag antibody in the THP-1 cells transfected Myc-Nur77 or vector. Statistical significance was determined using a two-tailed, unpaired Student's *t*-test. **P < 0.01.

A20, a ubiquitin-editing enzyme, has emerged as a critical NF-κB-negative regulator that functions by removing ubiquitin chains from the NF-kB essential transducer TRAF6 [14, 15]. We investigated the molecular mechanism by which Nur77 negatively regulates poly (I:C)-triggered NF-κB activation and found that overexpression of Nur77 significantly enhanced A20 expression at both mRNA (Figure 4A, left) and protein (Figure 4A, right) levels. Conversely, the mRNA (Figure 4B, left) and protein (Figure 4B, right) levels of A20 were more greatly attenuated in peritoneal macrophages from Nur77^-/-^ mice than in those from Nur77^+/+^ mice, suggesting that Nur77 is involved in the regulation of A20 expression. In addition, results further indicated that overexpression of Nur77 markedly enhanced the expression of A20 induced by poly (I:C) (Figure 4C). Similarly, poly (I:C) strongly induced A20 expression in peritoneal macrophages isolated from wild-type mice but not in Nur77^-/-^ mice (Figure 4D). This was consistent with *in vivo* observations of the increased expression of A20 in liver tissues of wild-type mice compared to those of Nur77^-/-^ mice after poly (I:C)/D-GalN injection (Figure 4E). These results indicated that Nur77 is indispensable to the expression of A20 induced by poly (I:C).

Given the fact that Nur77, like other nuclear receptors, functions as a transcriptional factor to modulate the expression of its target gene through binding to their promoter regions [22–24]. We speculated that Nur77 might bind A20 promoter region to enhance its expression. To test this hypothesis, we cloned part of the A20 promoter between -2000 bp and -1 bp to a luciferase reporter system. The results showed that overexpression of Nur77 significantly enhanced the luciferase activity of A20 promoter in a dose-dependent manner (Figure 4F). We performed further chromatin immunoprecipitation (ChIP) assays to determine the ability of Nur77 protein to bind to A20 promoter. The results revealed that Nur77 was enriched at A20 promoter region in Nur77-overexpresssed cells but not in vector-overexpressed control cells (Figure 4G). These data indicate that Nur77 enhances A20 expression through binding to A20 promoter.

### Nur77 suppresses NF-κB activation through a A20-dependent pathway

**Figure 5:**
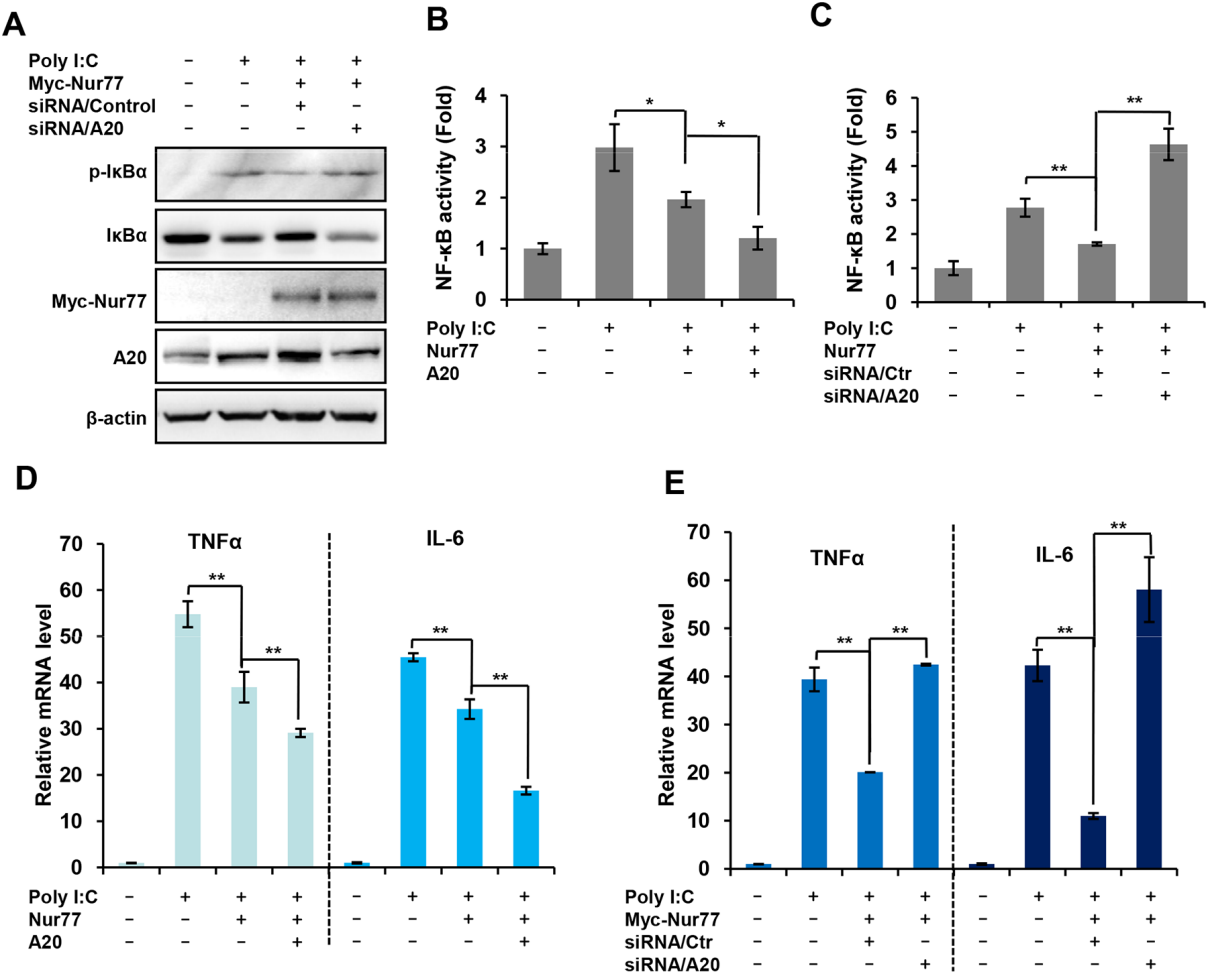
Inhibitory effect of Nur77 on poly (I:C)-activated NF-κB is dependent on A20 expression **(A)** Western blot analysis of the indicated protein in A20-sliencing RAW264.7 cells treated with vehicle or poly (I:C) (20 μg/ml). **(B, C)** Luciferase reporter assay for measuring NF-κB activity in Nur77-overexpressing RAW264.7 cells **(B)** or A20-sliencing RAW264.7 cells **(C)** treated with vehicle or poly (I:C) (20 μg/ml). Statistical significance was determined using a two-tailed, unpaired Student’s *t*-test. *P <0.05 and **P < 0.01. **(D, E)** qPCR assay of the indicated cytokine mRNA expression in Nur77-overexpressed RAW264.7 cells **(D)** or A20-sliencing RAW264.7 cells **(E)** treated with vehicle or poly (I:C) (20 μg/ml). Statistical significance was determined using a two-tailed, unpaired Student’s *t*-test. **P < 0.01.

The data given above indicate that A20 is a novel target gene of Nur77. We next addressed whether the regulation of NF-κB by Nur77 is dependent on A20. Western blot analysis revealed that overexpression of Nur77 significantly inhibited poly (I:C)-induced phosphorylation and degradation of IκBα, which was reversed to a considerable extent by silencing A20 expression in RAW264.7 cells (Figure 5A), suggesting that A20 is involved in Nur77-mediated NF-κB signaling. To further support this notion, we determined whether A20 is involved in the regulation of NF-κB activity and the regulation of the expression of its downstream target genes by Nur77. Luciferase reporter assays showed that overexpression of Nur77 substantially inhibited the activation of NF-κB induced by poly (I:C), which was greatly enhanced by A20 (Figure 5B). In contrast, knockdown of A20 reversed the effects that ectopic expression of Nur77 suppressed the activation of NF-κB induced by poly (I:C) (Figure 5C). Consistent with these results, the mRNA levels of the NF-κB downstream target genes TNF-α and IL-6 were further impaired by overexpression of A20 in Nur77-transfected RAW264.7 cells (Figure 5D). Conversely, expression of those inflammatory cytokines were markedly elevated when the expression of A20 was downregulated by siRNA in Nur77-transfected RAW264.7 cells (Figure 5E). Together, these results indicated that the inhibitory effects of Nur77 on the NF-κB activation are dependent on A20.

## DISCUSSION

Increasing amounts of evidence suggest that Nur77 plays protective roles in inflammatory disease. Nur77 functions as a protective factor through different mechanisms in different inflammatory diseases. For example, Nur77 prevents atherosclerosis via regulation of the polarization of macrophages [7], and it exerts a protective effect against inflammatory bowel disease by modulating Toll-like receptor (TLR) signaling [11]. Here, we found that Nur77 inhibits acute liver inflammation by transcriptionally regulating the ubiquitin-editing enzyme A20, which subsequently inhibits NF-κB activation.

Employing *in vivo* acute liver inflammation model in wild-type (Nur77^+/+^) and Nur77^-/-^ mice, we found that Nur77 deficiency resulted in acceleration of liver inflammation as characterized by increased inflammatory response and production of inflammatory cytokines and hepatocyte cell death. Consistently, Lack of Nur77 in mice was associated with increased risk of several inflammatory diseases including atherosclerosis, inflammatory bowel disease, and encephalitis [7, 10, 11]. In this way, these data indicate that Nur77 may act as a suppressor in inflammatory disease. We here further revealed that inflammatory signaling NF-κB activation caused by Nur77 deficiency in mice contributes to acute liver inflammation. Increasing numbers of studies have revealed that aberrant activation of NF-κB is a causative factor in the development of inflammatory diseases, and many proteins involve in regulation of this signaling [25–27]. In this study, we found that Nur77 is indeed involved in NF-κB signaling in poly (I:C)-induced liver inflammation. Ectopic expression of Nur77 greatly inhibited poly (I:C)-induced NF-κB activation. Conversely, silencing Nur77 expression in inflammatory cells markedly enhanced the activity of NF-κB induced by poly (I:C). Consistent with these findings, Nur77-knockout mouse models of acute liver inflammation showed elevated expression of pro-inflammatory cytokines. One important finding reported here is the implication of the ubiquitin-editing enzyme A20, a critical NF-κB-negative regulator, in the inhibition of NF-κB by Nur77. Overexpression of A20 increased markedly, whereas silencing A20 expression greatly impaired the inhibitory effect of Nur77 on NF-κB signaling. These results suggest a requirement for A20 in Nur77-mediated inflammatory signaling NF-κB.

Nur77 can act in the cytoplasm as a signal mediator to regulate inflammatory disease. For example, Nur77 functions as a negative regulator of TLR-IL-1R signaling via interaction with TRAF6 in the cytoplasm to protect against the development of inflammatory bowel disease and sepsis [11, 28]. However, Nur77 also functions as a transcriptional factor in the nucleus to regulate the expression of its target genes, including BRE, Survivin, and GLUT4 [29–31]. We recently reported that Nur77 could bind directly to the promoter of matrix metalloproteinase (MMP)-9 and colony stimulating factor 1 receptor (CSF-1R) to regulate their expression [23, 24]. Furthermore, we here found that A20 is a novel target gene of Nur77. Our results from luciferase and ChIP assay suggested that Nur77 induces A20 expression through binding to A20 promoter. However, we did not find a potential Nur77 binding site within the region of A20 promoter between -2000 bp and -1 bp. Thus, these results indicated that Nur77 indirectly binds to A20 promoter and induces its expression, which might be mediated by other molecules. The regulatory role of Nur77 on A20 expression is required for Nur77's anti-inflammatory effect in liver inflammation. In an *in vivo* acute liver inflammation model, the levels of A20 expression in liver tissues from wild-type mice were significantly higher than in Nur77^-/-^ mice. In this way, Nur77 can exert both transcriptional and extranuclear effects in inflammatory disease, depending on the pathophysiological conditions.

In summary, we here uncovered an important protective effect of Nur77 in poly (I:C)-induced acute liver inflammation. It takes place through regulation of the NF-κB inflammatory signaling pathway. We also found A20 to be a novel downstream target gene of Nur77. These findings highlight a new therapeutic strategy for liver inflammation, targeting Nur77.

## MATERIALS AND METHODS

### Antibody and regents

Lipofectamine 2000 and TRIZOL reagent were purchased from Invitrogen. Rabbit or mouse antibodies against A20, PARP and p-IκBα were purchased from Cell Signaling Technology. Rabbit or mouse antibodies against p65 and Myc-tag were purchased from Santa Cruz Biotechnology. Rabbit antibody against IκBα was purchased from Abcam. Mouse antibody against b-actin was purchased from Sigma-Aldrich, and WesternBright ECL regents were purchased from Advansta.

### Cell culture and transfection

RAW264.7 and THP-1 cells were obtained from the Cell Bank of the Chinese Academy of Sciences (Shanghai, China) and were maintained in RPMI 1640 containing 10% fetal bovine serum. These cells were transfected with the indicated vector or plasmid using Lipofectamin 2000.

### Isolation of peritoneal macrophage

Nur77^+/+^ or Nur77^-/-^ mice were intraperitoneally injected with 4% thioglycolate (2 ml). After 3 d, using ice-cold DMEM to wash the peritoneal cavity and the cells in the peritoneal exudates were isolated. Collected cells were incubated at 37°C in a humidified atmosphere of 5% CO2 and 95% air for 4 h, and adherent cells were taken as peritoneal macrophages.

### Cell lysis and fractionation

Cell lysates were extracted with radioimmunoprecipitation assay lysis buffer (50 mM Tris–HCl pH 7.4, 1% NP-40, 0.25% Na-deoxycholate, 150 mM NaCl) containing protease inhibitor (Roche). For cellular fractionation, cytoplasmic fraction was purified with cold buffer containing 10mM HEPES-KOH (pH 7.9), 1.5 mM MgCl_2_, 10mM KCl and 0.5 mM DTT. whereas nuclear proteins were prepared by resuspended the pellets in cold high-salt buffer containing 20mM HEPES-KOH (pH 7.9), 25% glycerol, 420 mM NaCl, 1.5 mM MgCl_2_, 0.2 mM EDTA and 0.5 mM DTT.

### Western blot

Western blot was performed as recently described [23, 32]. Briefly, whole cell extracts were prepared by lysing the cells in ice-cold lysis buffer (50 mM Tris–HCl pH 7.4, 1% NP-40, 0.25% Na-deoxycholate, 150 mM NaCl) containing protease inhibitor (Roche). Equal amount of proteins were electrophoresed on 8%-10% SDS-PAGE gel, and then the divided proteins were transferred to a PVDF membrane (Millipore). Antibodies against the indicated protein were used to detected their expression.

### RNA extraction and qPCR analysis

Total RNA were extracted using Trizol LS (Invitrogen), and cDNA was synthesized using RevertAid™ First Strand cDNA Synthesis Kits (Fermentas). qPCR was performed using Power SYBR® Green PCR Master Mix (TaKaRa, Japan). Normalization was performed with β-actin. Specific primers with the following sequences were used: A20 (Human), forward 5'-GTCCGGAAGCTTGTGGCGCT-3' and reverse 5'-CCAAGTCTGTGTCCTGAACGCCC-C-3'; A20 (Mouse), forward 5'-CAGTGGGAAGGGACACAACT-3' and reverse 5'-GCAGTGGCAGAAACTTCCTC-3'; β-actin (Human) forward 5'-CACCAACTGGGACGACATG-3' and reverse 5'- GCACAGCCTGGATAGCAA-C-3'; β-actin (Mouse) forward 5'-TGGAATCCTGTGGCATCCATGAAAC-3' and reverse 5'- TAAAACGCAGCTCAGTAACAGTCCG-3'.

### Luciferase reporter assay

RAW264.7 Cells were transfected with the pGL3-A20-promoter-Luc reporter constructs, β-galactosidase (β-gal) expression vectors and other relevant plasmids. The luciferase activity was determined after transfection for 36 h. β-gal activity served as internal control.

### Chromatin immunoprecipitation (Chip)

RAW264.7 cells were harvested after transfection with Myc-Nur77 or vector for 36 h. The Chip experiment was performed using SimpleChIP plus Enzymatic Chromatin IP kit (Cell Signaling Technology). Briefly, cells were crosslinked with 37% formaldehyde and quenched with glycine. Cross-linked chromatin was digested by micrococcal nuclease and subjected to immunoprecipitation with specific antibody against Myc-tag. The protein/DNA complexes were precipitated with protein A beads and digested with proteinase K to remove proteins. The specific DNA fragments were subjected to PCR amplification using the following primers: forward 5'- GAGGATTGAAGTCATGGAGC-3' and reverse 5'-GTCTTGGTTTCACCTTCAGG-3'

### Animal studies

Nur77^-/-^ mice on a C57 background were obtained from the Jackson Laboratories. All mice were housed in clean and comfortable animal rooms at the Laboratory Animal Centre in Soochow University (China). For acute liver inflammation model, Nur77^+/+^ and Nur77^−/−^ mice (8-10 weeks, male) were intraperitoneally coinjected with poly (I:C) (6.25mg/kg) and D-GalN (500mg/kg). 5 h later, mice were anesthetized with ether and bled from the eye. The concentration of alanine aminotransferase (ALT), aspartate aminotransferase (AST), TNFα and IL-6 in blood were determined by ELISA. At the same time, RNA was extracted from liver tissue and relative mRNA level of TNFα, IL-6, IL-12 and IFN-β were measured by qPCR. Animal procedures were approved by the Animal Care and Use Committee of Soochow University.

### Statistical analysis

Each assay was performed in three independent experiments. Data were presented as mean ± s.d. The Student *t* test (unpaired, two-tailed) was used to compare two groups of independent samples. One-way ANOVA was used for multiple comparisons. *p* < 0.05 was considered statistically significant.

## Abbreviations

TNFα: tumor necrosis factor alpha
IL-6: interleukin-6
IL-12: interleukin-12
IFN-β: interferon beta
MCP-1: monocyte-chemoattractant protein-1
qPCR: quantitative PCR
Chip: Chromatin immunoprecipitation

## References

[1] Zimmermann HW, Trautwein C, Tacke F (2012). Functional role of monocytes and macrophages for the inflammatory response in acute liver injury. Front Physiol.

[2] Nakamoto Y, Kaneko S (2003). Mechanisms of viral hepatitis induced liver injury. Curr Mol Med.

[3] Higuchi H, Gores GJ (2003). Mechanisms of liver injury: an overview. Curr Mol Med.

[4] Lee SO, Li X, Khan S, Safe S (2011). Targeting NR4A1 (TR3) in cancer cells and tumors. Expert Opin Ther Targets.

[5] To SK, Zeng JZ, Wong AS (2012). Nur77: a potential therapeutic target in cancer. Expert Opin Ther Targets.

[6] Zhang XK (2007). Targeting Nur77 translocation. Expert Opin Ther Targets.

[7] Hanna RN, Shaked I, Hubbeling HG, Punt JA, Wu R, Herrley E, Zaugg C, Pei H, Geissmann F, Ley K, Hedrick CC (2012). NR4A1 (Nur77) deletion polarizes macrophages toward an inflammatory phenotype and increases atherosclerosis. Circ Res.

[8] Li L, Liu Y, Chen HZ, Li FW, Wu JF, Zhang HK, He JP, Xing YZ, Chen Y, Wang WJ, Tian XY, Li AZ, Zhang Q (2015). Impeding the interaction between Nur77 and p38 reduces LPS-induced inflammation. Nat Chem Biol.

[9] Kurakula K, Vos M, Logiantara A, Roelofs JJ, Nieuwenhuis MA, Koppelman GH, Postma DS, van Rijt LS, de Vries CJ (2015). Nuclear Receptor Nur77 Attenuates Airway Inflammation in Mice by Suppressing NF-κB Activity in Lung Epithelial Cells. J Immunol.

[10] Shaked I, Hanna RN, Shaked H, Chodaczek G, Nowyhed HN, Tweet G, Tacke R, Basat AB, Mikulski Z, Togher S, Miller J, Blatchley A, Salek-Ardakani S (2015). Transcription factor Nr4a1 couples sympathetic and inflammatory cues in CNS-recruited macrophages to limit neuroinflammation. Nat Immunol.

[11] Wu H, Li XM, Wang JR, Gan WJ, Jiang FQ, Liu Y, Zhang XD, He XS, Zhao YY, Lu XX, Guo YB, Zhang XK, Li JM (2016). NUR77 exerts a protective effect against inflammatory bowel disease by negatively regulating the TRAF6/TLR-IL-1R signalling axis. J Pathol.

[12] Sekiya T, Kashiwagi I, Yoshida R, Fukaya T, Morita R, Kimura A, Ichinose H, Metzger D, Chambon P, Yoshimura A (2013). Nr4a receptors are essential for thymic regulatory T cell development and immune homeostasis. Nat Immunol.

[13] You B, Jiang YY, Chen S, Yan G, Sun J (2009). The orphan nuclear receptor Nur77 suppresses endothelial cell activation through induction of IkappaBalpha expression. Circ Res.

[14] Shembade N, Ma A, Harhaj EW (2010). Inhibition of NF-kappaB signaling by A20 through disruption of ubiquitin enzyme complexes. Science.

[15] Boone DL, Turer EE, Lee EG, Ahmad RC, Wheeler MT, Tsui C, Hurley P, Chien M, Chai S, Hitotsumatsu O, McNally E, Pickart C, Ma A (2004). The ubiquitin-modifying enzyme A20 is required for termination of Toll-like receptor responses. Nat Immunol.

[16] Xia M, Liu J, Wu X, Liu S, Li G, Han C, Song L, Li Z, Wang Q, Wang J, Xu T, Cao X (2013). Histone methyltransferase Ash1l suppresses interleukin-6 production and inflammatory autoimmune diseases by inducing the ubiquitin-editing enzyme A20. Immunity.

[17] Yuk JM, Kim TS, Kim SY, Lee HM, Han J, Dufour CR, Kim JK, Jin HS, Yang CS, Park KS, Lee CH, Kim JM, Kweon GR (2015). Orphan Nuclear Receptor ERRα Controls Macrophage Metabolic Signaling and A20 Expression to Negatively Regulate TLR-Induced Inflammation. Immunity.

[18] Murakawa Y, Hinz M, Mothes J, Schuetz A, Uhl M, Wyler E, Yasuda T, Mastrobuoni G, Friedel CC, Dölken L, Kempa S, Schmidt-Supprian M, Blüthgen N (2015). RC3H1 post-transcriptionally regulates A20 mRNA and modulates the activity of the IKK/NF-κB pathway. Nat Commun.

[19] Baranova IN, Souza AC, Bocharov AV, Vishnyakova TG, Hu X, Vaisman BL, Amar MJ, Chen Z, Kost Y, Remaley AT, Patterson AP, Yuen PS, Star RA, Eggerman TL (2016). Human SR-BI and SR-BII Potentiate Lipopolysaccharide-Induced Inflammation and Acute Liver and Kidney Injury in Mice. J Immunol.

[20] Yan TD, Wu H, Zhang HP, Lu N, Ye P, Yu FH, Zhou H, Li WG, Cao X, Lin YY, He JY, Gao WW, Zhao Y (2010). Oncogenic potential of retinoic acid receptor-gamma in hepatocellular carcinoma. Cancer Res.

[21] O’Dea E, Hoffmann A (2009). NF-κB signaling. Wiley Interdiscip Rev Syst Biol Med.

[22] Zhan YY, He JP, Chen HZ, Wang WJ, Cai JC (2013). Orphan receptor TR3 is essential for the maintenance of stem-like properties in gastric cancer cells. Cancer Lett.

[23] Wang JR, Gan WJ, Li XM, Zhao YY, Li Y, Lu XX, Li JM, Wu H (2014). Orphan nuclear receptor Nur77 promotes colorectal cancer invasion and metastasis by regulating MMP-9 and E-cadherin. Carcinogenesis.

[24] Li XM, Wang JR, Shen T, Gao SS, He XS, Li JN, Yang TY, Zhang S, Gan WJ, Li JM, Wu H (2017). Nur77 deficiency in mice accelerates tumor invasion and metastasis by facilitating TNFα secretion and lowering CSF-1R expression. PLoS One.

[25] Lin TH, Pajarinen J, Lu L, Nabeshima A, Cordova LA, Yao Z, Goodman SB (2017). NF-κB as a Therapeutic Target in Inflammatory-Associated Bone Diseases. Adv Protein Chem Struct Biol.

[26] Pasparakis M (2009). Regulation of tissue homeostasis by NF-kappaB signalling: implications for inflammatory diseases. Nat Rev Immunol.

[27] Curran CL, Blackwell TS, Christman JW (2001). NF-kappaB: a therapeutic target in inflammatory diseases. Expert Opin Ther Targets.

[28] Li XM, Zhang S, He XS, Guo PD, Lu XX, Wang JR, Li JM, Wu H (2016). Nur77-mediated TRAF6 signalling protects against LPS-induced sepsis in mice. J Inflamm (Lond).

[29] Liu JJ, Zeng HN, Zhang LR, Zhan YY, Chen Y, Wang Y, Wang J, Xiang SH, Liu WJ, Wang WJ, Chen HZ, Shen YM, Su WJ (2010). A unique pharmacophore for activation of the nuclear orphan receptor Nur77 in vivo and in vitro. Cancer Res.

[30] Lee SO, Andey T, Jin UH, Kim K, Singh M, Safe S (2012). The nuclear receptor TR3 regulates mTORC1 signaling in lung cancer cells expressing wild-type p53. Oncogene.

[31] Chao LC, Zhang Z, Pei L, Saito T, Tontonoz P, Pilch PF (2007). Nur77 coordinately regulates expression of genes linked to glucose metabolism in skeletal muscle. Mol Endocrinol.

[32] Guo PD, Lu XX, Gan WJ, Li XM, He XS, Zhang S, Ji QH, Zhou F, Cao Y, Wang JR, Li JM, Wu H (2016). RARγ Downregulation Contributes to Colorectal Tumorigenesis and Metastasis by Derepressing the Hippo-Yap Pathway. Cancer Res.

